# Effect of Homogenized Callus Tissue on the Rheological and Mechanical Properties of 3D-Printed Food

**DOI:** 10.3390/gels10010042

**Published:** 2024-01-04

**Authors:** Elena Dushina, Sergey Popov, Andrey Zlobin, Ekaterina Martinson, Nikita Paderin, Fedor Vityazev, Kseniya Belova, Sergey Litvinets

**Affiliations:** 1 Institute of Biology and Biotechnology, Vyatka State University, 36, Moskovskaya Str., 610000 Kirov, Russia; usr21537@vyatsu.ru (E.D.); usr00138@vyatsu.ru (A.Z.); biotech.vgu@gmail.com (E.M.); kd_koposova@vyatsu.ru (K.B.); litvinets@list.ru (S.L.); 2 Institute of Physiology of Federal Research Centre “Komi Science Centre of the Urals Branch of the Russian Academy of Sciences”, 50, Pervomaiskaya Str., 167982 Syktyvkar, Russia; paderin_nm@mail.ru (N.P.); rodefex@mail.ru (F.V.)

**Keywords:** lupin, callus tissue, agar, mashed potatoes, 3D food printing, gel properties, texture, rheology

## Abstract

The aim of the study was to develop ink enriched with a high content of lupine callus tissue (CT) suitable for 3D printing. Printable ink obtained using mashed potatoes (20 g/100 mL) and a 3% agar solution was used as the parent CT-free ink (CT0). Viscosity increased from 9.6 to 75.4 kPa·s during the cooling of the CT0 ink from 50 to 20 °C, while the viscosity of the ink with 80 g/100 mL of CT (CT80) increased from 0.9 to 5.6 kPa·s under the same conditions. The inclusion of CT was shown to decrease the hardness of 3D-printed food gel from 0.32 ± 0.03 to 0.21 ± 0.03 N. The storage modulus G’ value was 7.9 times lower in CT80 samples than in CT0 samples. The values of fracture stress for CT80 and CT0 inks were 1621 ± 711 and 13,241 ± 2329 Pa, respectively. The loss tangent and the limiting strain did not differ in CT0 and CT80, although the value of the fracture strain was 1.6 times higher in the latter. Thus, the present study demonstrates that CT may be added to printing ink in order to enhance food with plant cell material and enable the 3D printing of specially shaped foods.

## 1. Introduction

Foods derived from plants are critical to a balanced diet and are becoming more and more well liked as research on their benefits to human health expands [1]. However, providing plant food to the world’s ever-increasing population is becoming increasingly difficult [2]. To provide a variety of nutritious plant-based foods, new technologies must be created. One potential technology in this regard is the culture of plant cells as a novel method of producing plant-based food. Plant cell cultures are currently being used in the production of several food components that have been registered [3,4]. Many important nutrients can be obtained through the use of cell cultures; these possibilities have been thoroughly investigated, and methods for enhancing food with health-promoting components have been devised. 

Three-dimensional (3D) printing enables the creation of complex geometric shapes in the individual manufacturing of dishes based on the individual’s specific nutritional demands and calorie consumption [5]. Food 3D printing offers a new and exciting possibility for those with specific nutritional needs, including youngsters, the elderly, patients with feeding or swallowing difficulties, and individuals with a variety of inflammatory and metabolic problems. This technique is simple to use, enables large manufacturing, and reduces food waste and transportation and storage expenses, all of which benefit the economy and the environment [6]. Essentially, the extrusion method is used to 3D print food products. This process involves putting a liquid or semi-solid substance through a nozzle and layer by layer building a three-dimensional structure based on the digital model. Food-printing materials may include mashed potatoes, chocolate, cheese, pork, surimi, vegetable, and fruit pastes [7]. Importantly, not only single materials can be 3D printed but also multi-ingredient ink formulations, as shown in the recent study [8]. However, with extrusion printing, a programmed 3D design is not always attainable. The physicochemical characteristics of the food-grade inks (particular mechanisms of aggregation and gelation, water retention capacity, and rheological and thermal properties) are the key determinant of the quality of the printed product [9]. 

Personalized nutrition is one of the most exciting promises of biotechnological production of plant raw materials for food and 3D printing food technology, since both approaches can be aimed at creating a diet enriched with special health-promoting components for specific population groups [3,4,5,10]. However, there has been minimal research into the use of plant cell cultures in 3D printing, despite the fact that the number of original studies on plant-based 3D-printable materials has expanded dramatically in recent years [11]. The impact of adding plant cell cultures on the rheological qualities of food ink and how this affects the final 3D product’s mechanical and structural attributes remains partially unknown. 

In our previous study [12], food ink made from 3% k-carrageenan and callus tissue of lupin (*L. angustifolius*) was developed, and its printability was evaluated. The results demonstrated that the ink enriched with lupin calluses LA14 and LA16 was successfully 3D printed at concentrations of 33 and 50 g/100 mL, respectively. It was shown that the residual content of 2,4-dichlorophenoxyacetic acid in LA14 callus was too high to be acceptable under food safety requirements. Therefore, LA16 callus can only be considered a promising food ink ingredient. It should be noted that food ink made from 3% k-carrageenan was successfully 3D printed only with a low degree of enrichment with callus tissue, while the ink was cured in the printer extruder at callus concentrations of more than 33 g/100 mL [12]. Additionally, print accuracy was not assessed, which was a limitation of the study. The rheological properties of the callus biomass used to obtain callus tissue were not determined, which was another gap in the earlier work. It can be assumed that rheological and structural–mechanical properties are required to investigate the changes in callus tissue properties during the preparation process before it is incorporated into the ink composition. Therefore, further modification of the callus ink formulation appears to be required to improve 3D printability and the development of 3D food gels enriched with plant material. In the present study, we hypothesized that formulations based on agar solution and mashed potatoes (MPs) would be suitable for 3D food inks, which can be enriched with callus tissue without reducing printability. Agar is a hydrocolloid produced from marine red algae that is widely utilized in the culinary, food, and confectionery sectors due to its ability to form strong thermo-reversible gels in aqueous solutions [13]. Importantly, agars, like other seaweed polysaccharides, are valuable as dietary fiber because the human digestive system hardly absorbs them [14,15,16,17]. Several studies have used agar as a component of 3D-printing inks [18,19]. Contrary, MP is widely used in 3D food printing as it has good extrusion ability and load-bearing capacity without deformation [18,20,21]. Although MP is itself extrudable, the use of additives (e.g., alginate, agar, or gums such as xanthan and k-carrageenan) in concentrations as low as 1% has been found to improve rheological properties and print outcomes such as texture, surface quality, and shape stability [22]. The starch contained in MP could presumably help reduce the separation of the liquid phase from aqueous callus tissue during extrusion.

The aim of the study was to develop ink enriched with a high content of lupine callus tissue suitable for 3D printing using agar and MP.

## 2. Results

### 2.1. Callus Characterization

**Figure 1:**
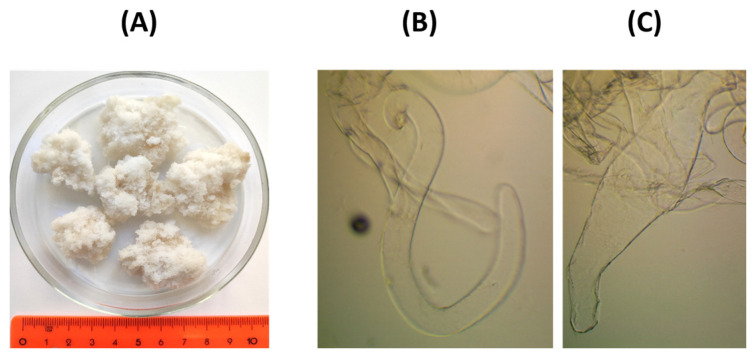
Appearance of the lupin callus lumps (**A**) and micrographs (magnification 10×) of the S-shaped (**B**) and spindle-shaped (**C**) callus cells.

Lupine callus was grown in Petri dishes on Murashige–Skoog medium in the form of light-colored loose lumps with an average volume of 3.6 ± 0.9 cm^3^ and a weight of 3.2 ± 0.6 g (Figure 1A). Callus biomass was formed by cells of elongated and curved shape, often S-shaped, less often spindle-shaped, with an average width of 61 ± 8 µm and an average length of 986 ± 205 µm (Figure 1B,C).

Fresh callus biomass was frozen for the accumulation and subsequent use in a food gel composition. General characteristics of callus thawed after freezing are presented in Table 1 in comparison with fresh callus biomass. The weight, volume, and density of callus biomass decreased after freezing and thawing, probably due to water loss, as the moisture content decreased by 1.9%. In addition, the frozen/thawed callus had a lower pH.

**Figure 2:**
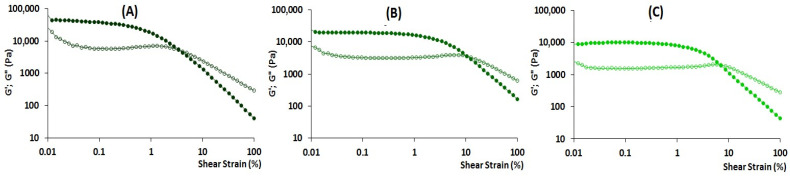
Rheological properties of fresh callus biomass (**A**), frozen/thawed callus biomass (**B**), and homogenized callus tissue CT (**C**): storage modulus (G′, filled symbols) and loss modulus (G″, empty symbols) test results are represented as a function of shear strain.

The rheological characteristics of fresh and frozen/thawed callus biomass were obtained using small-deformation dynamic rheological measurements (Figure 2). Strain sweep experiments were performed from 0.01 to 100% strain amplitude using a controlled shear rate mode at 20 °C at a constant frequency. The storage modulus G′ of both fresh and frozen/thawed callus biomass was greater than the loss modulus G″ throughout the linear (LVE) viscoelastic region (Figure 2A,B). The obtained high G′_LVE_ value and the loss tangent (tan δ) in LVE < 1.0 demonstrated that fresh and frozen/thawed callus biomass had the properties of a strong physical gel. However, G′_LVE_, G″_LVE_, and G*_FP_ values for the frozen/thawed callus were 39, 47, and 34% lower than those for the fresh one. The limiting strain (γL) at which G′ sharply diminished with the increase in strain and the fracture strain (γFr) were 2-fold and 2.8 times higher for frozen/thawed callus than those for fresh ones. The values of limiting stress (τL) measured at the fracture point were 24,917 ± 1359 and 18,171 ± 4058 Pa (*p* < 0.05) for the fresh and frozen/thawed callus, respectively. The values of fracture stress (τFr) measured at the fracture point were 4428 ± 971 and 3494 ± 527 Pa (*p* < 0.05) for the fresh and frozen/thawed callus, respectively. The slope of the loss tangent after flow point (tan [δ]_AF_) was lower for frozen/thawed than for fresh callus biomass. The rheological measurements at the frequency sweep tests for both fresh and frozen/thawed callus showed that G′ > G″ both times at the stress level of 9 Pa. The frozen/thawed callus had a lower network extension (z) value than the fresh callus (Table 2).

In a preliminary study on the preparation of ink for 3D printing, it was found that both fresh and frozen/thawed callus biomass were not suitable for 3D printing. In particular, fresh callus cells are mechanically destructed by the pressure of the piston in the extruder with the subsequent release of liquid from the nozzle, while large aggregates of cell wall debris completely clog the extruder nozzle. The biomass of frozen/thawed callus consists of solid explants measuring 1.5–2 cm, which also do not pass through the 3 mm nozzle of the extruder, while the piston squeezes out only the liquid part of the loaded biomass. Similarly, extrusion was not possible when either fresh or frozen/thawed callus was added to the agar solution and/or mashed potato puree.

Therefore, the frozen/thawed callus biomass was then homogenized to obtain homogenized callus tissue (CT) for inclusion in the formulation for 3D printing ink. CT also showed G′ >> G″, although the values of both moduli were significantly lower than those of frozen/thawed callus biomass (Figure 2C). Homogenization was found to reduce γL, τL, γFr, and τFr values (Figure 2C). The values of *n*′, *n*″, and z obtained from the frequency test were the same for frozen/thawed callus biomass and CT.

### 2.2. Composition of CT

The soluble solid content of the CT was 4.3%, while the content of protein, fat, and carbohydrates was 0.28, 4.3, and 45.5%, respectively. The main free monosaccharides in CT were glucose (Glc) and fructose residues, the content of which was 5.1% and 9.8% of soluble solid content, respectively. In addition, free arabinose (Ara), rhamnose (Rha), xylose (Xyl), mannose (Man), and galactose (Gal) residues were identified in trace amounts. CT was found to contain two main polysaccharide fractions of the total yield of 8.6% of dry tissue weight (Table 3). The main components of the first polysaccharide fraction (LA-1) were uronic acid (UA) residues with a degree of methyl esterification of 10.1% as well as Gal and Ara residues. In addition, it contained residues of Glc, Xyl, Rha, and Man. The main component of the second polysaccharide fraction (LA-2) was UA residues with a degree of methyl esterification of 5.8%. Using the GLC-MS method of trimethylsilyl ethers, galacturonic acid residues, as well as trace amounts of glucuronic acid residues, were identified in LA-1 and LA-2. 

The yield of the free lipid fraction of lupine callus in terms of dry tissue weight was 4.3%. Unsaturated fatty acids in the composition of free callus lipids accounted for 58.4%. Among them, linoleic acid predominated (42.9%). The main saturated fatty acid was palmitic acid (32.1%).

Energy-dispersive X-ray spectroscopy (EDS) analysis revealed contents of calcium and magnesium (8.72 and 3.91 wt%, respectively) in the CT.

### 2.3. Effect of CT on the 3D Printability of Food Ink

**Figure 3:**
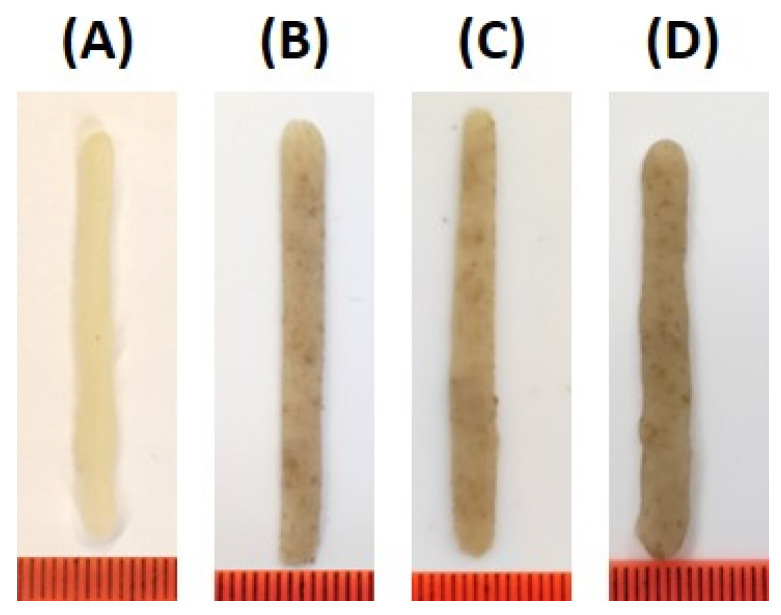
Representative results of the printing of 1D lines using CT0 (**A**), CT80 (**B**), CT85 (**C**), and CT75 (**D**) inks.

**Figure 4:**
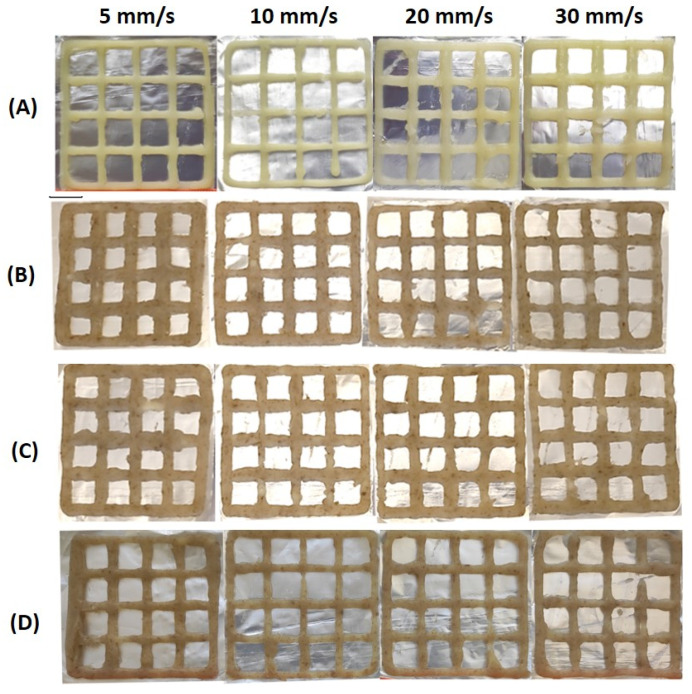
Representative results of using CT0 (**A**), CT80 (**B**), CT85 (**C**), and CT75 (**D**) inks for printing a 2D grid with velocities of 5, 10, 20, and 30 mm/s.

**Figure 5:**
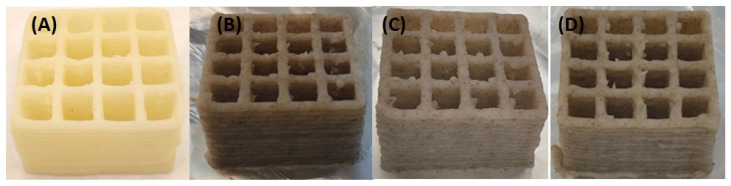
Representative results of the printing of 3D cubic structure using CT0 (**A**), CT80 (**B**), CT85 (**C**), and CT75 (**D**) inks.

**Figure 6:**
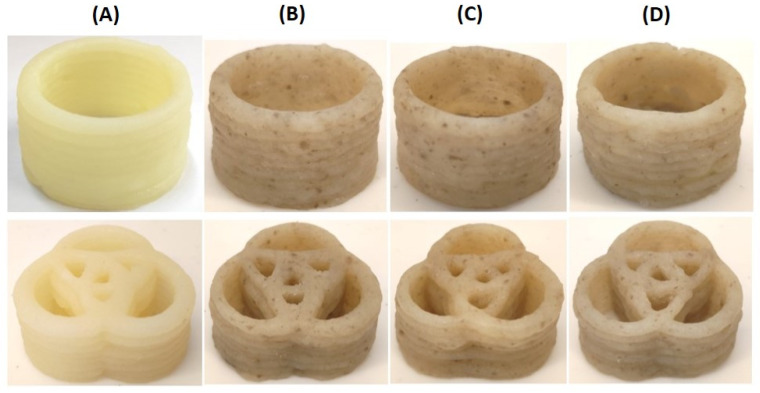
Representative results of the printing of 3D food gels with rounded shapes using CT0 (**A**), CT80 (**B**), CT85 (**C**), and CT75 (**D**) inks.

Parent ink formulation (CT0) was obtained using MP (20 g/100 mL) and a 3% agar solution and contained no CT. CT0 ink had good printability, allowing for the printing of 1D lines (Figure 3A), 2D grids (Figure 4A), 3D cubic structures (Figure 5A), and 3D objects with rounded shapes (Figure 6A).

**Figure 7:**
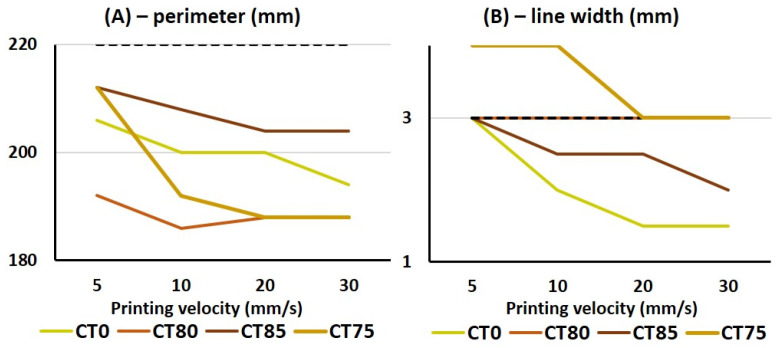
The square perimeter (**A**) and line width (**B**) of a 2D grid printed using CT0, CT80, CT85, and CT75 inks in comparison with the sizes of the target model grid (black dotted line).

CT was included in the CT0 ink formulation in varying proportions with MP. A 2D grid was printed using the ink formulations CT0, CT80, CT75, and CT85 in order to evaluate the accuracy of the printing. The square perimeter and line width of the target model grid were set to 220 and 3 mm, respectively. As shown in Figure 4, all inks allowed us to print a grid similar to the target model. However, the dimensions of the printed samples differed significantly from those specified (Figure 7). Printing accuracy with CT0 ink gradually decreased from 93 to 88% from the specified perimeter line with increasing printing speed. The line width in the CT0 grid was significantly smaller than that in the model grid at printing speeds above 5 mm/s. The accuracy of printing a perimeter line using CT80 ink was 85–87%, while the line thickness was 100% consistent with the specified model.

Differences in the printability of inks with different CT contents appeared when printing a 3D cubic structure, which was a shape formed from fifteen 2D grids superimposed on each other. The target cubic structure was 3D printed using CT80 ink, in which the content of CT was equal to 80 g/100 mL (Figure 5B). The CT85 and CT75 inks, in which the proportion of CT in the MP:CT ratio was increased or decreased by 5 g (MP:CT content of 15:85 and 25:75 g/100 mL, respectively), showed acceptable printability as well (Figure 5C,D). However, inks with a MP:CT content of 80:20, 60:40, and 40:60 g/100 mL failed to allow 3D printing because the ink quickly thickened in the 3D printer extruder. Inks containing no MP as well as those consisting only of CT (50–100 g/100 mL) and 3% agar were unprintable as described above. The good printability of CT0, CT80, CT85, and CT75 inks was also demonstrated when printing 3D food gels with rounded shapes (Figure 6).

### 2.4. Effect of CT on the Properties of the 3D-Printed Food Gels

**Figure 8:**
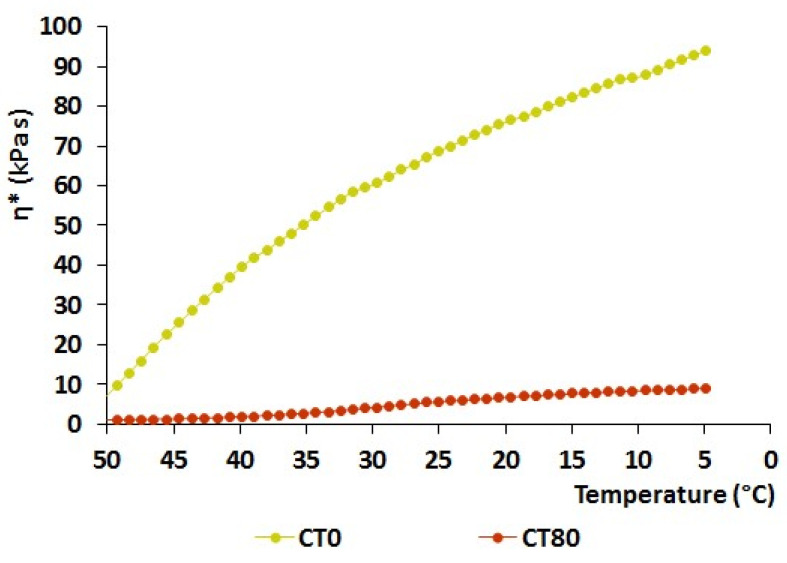
Complex viscosity at temperature cooling (rate: 5 °C/min from 50 to 5 °C) ramp tests performed at constant frequency (1.0 Hz) for CT0, CT75, and CT85 inks.

The effect of the inclusion of CT on the gelling properties of ink composition was firstly observed during the cooling of the ink from 50 to 20 °C at a temperature sweep of 5 °C/min. Viscosity increased from 9.6 to 75.4 kPa·s during the cooling of the CT0 ink from 50 to 20 °C, while the viscosity of the CT80 ink increased from 0.9 to 5.6 kPa·s under the same conditions. Thus, the viscosity of CT0 ink was 13.5 times higher than that of CT80 ink at a temperature of 20 °C (Figure 8).

Samples measuring 2 × 2 × 2 cm were printed to determine the mechanical and rheological properties of 3D food gels. The mechanical behavior of 3D-printed food gels was first monitored in the puncture test where all gels demonstrated force–distance curves with one peak. Hardness decreased in CT80 3D-printed food gels compared to CT0, indicating less stability of the CT80 gel networks under large deformation. The Young’s modulus of the CT80 3D-printed food gel was 30% lower than those of the CT0 3D-printed gel (Table 4). The elasticity of the CT80 3D-printed food gel increased relative to the level of the CT0 3D-printed food gel.

The strain sweep test (1 Hz, 9.0 Pa) yielded rheological characteristics representing the strength of linkage, number of linkages, and timescale of the junction zone in the 3D-printed food gels (Table 5). According to Table 5A, the parameters G′_LVE_, G*_LVE_, and tan [δ]_AF_ were 7.9, 7.6, and 3.4 times lower in CT80 than in CT0, indicating that the inclusion of CT to the ink decreased the strength of linkage in 3D-printed samples. The number of linkages in 3D-printed CT80 food gel seemed to be lower than in CT0, as indicated by the lower τFr value (Table 5B). Parameters tan [δ]_LVE_ and γL that concern the timescale of the junction zone did not differ in CT0 and CT80, although the value of γFr was 1.6 times higher in the latter (Table 5B).

**Figure 9:**
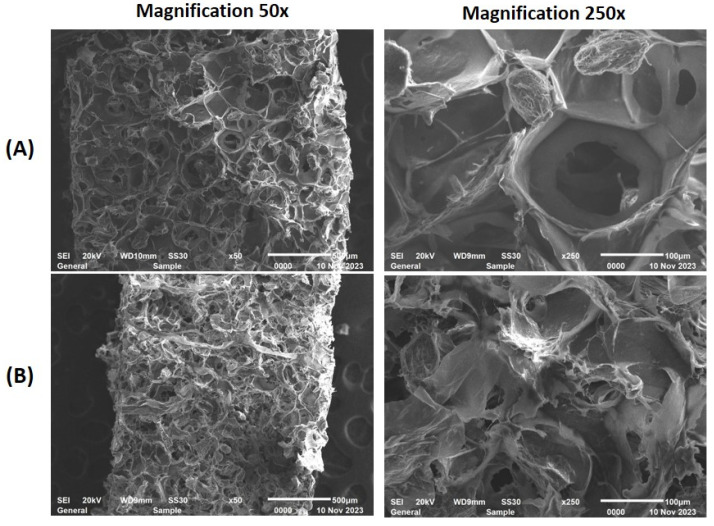
Scanning electron micrographs of the 3D-printed food gels prepared using CT0 (**A**) and CT80 (**B**) inks at magnification 50× (left column) and 250× (right column).

Figure 9 shows the morphology of CT0 and CT80 3D-printed food gels. Gel 3D-printed with CT0 ink formed a spongy network structure consisting of clearly visible polygonal pores. The internal microstructure of the gel 3D-printed with CT80 ink was apparently denser with smaller amorphous pores than that of the CT0 sample. Whole callus cells were not detected on the CE-containing sample.

### 2.5. Comparison of the Properties of the 3D-Printed and Molded Food Gels

Molded food gels were prepared by pouring CT0 and CT80 inks at a temperature of 50 °C into silicone molds (2 × 2 × 2 cm) and cooling them to room temperature to study the effect of the 3D-printing procedure on the properties of the sample. The hardness of the 3D-printed CT0 food gel was 16% lower than those of the molded CT0 one (Table 3). The Young’s modulus of CT0 and CT80 3D-printed samples was found to be 55 and 45% lower than those of molded samples of the corresponding composition. In contrast, the 3D-printing procedure seemed to enhance the elasticity of CT0 and CT80 food gels (Table 4).

**Figure 10:**
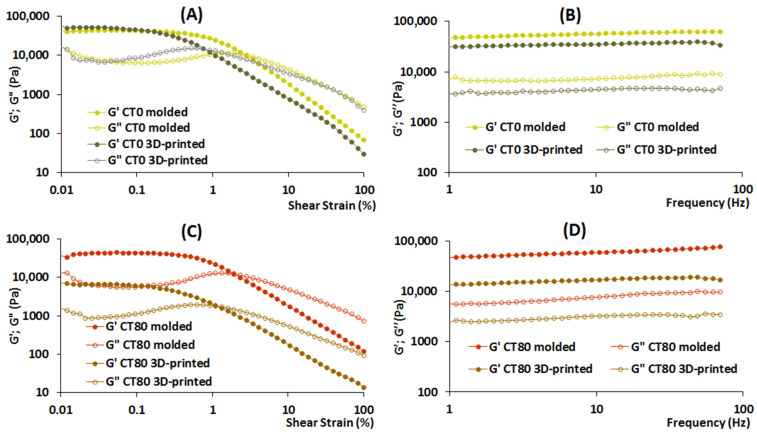
Rheological properties of 3D-printed and molded gels: storage modulus (G′, filled symbols) and loss modulus (G″, empty symbols) test results are represented as a function of shear strain (**A**,**C**) or frequency (**B**,**D**).

The differences in rheological characteristics obtained using strain sweep and frequency sweep experiments also demonstrated the influence of the 3D-printing procedure on the properties of the resulting gels (Figure 10). The values of G′ and G″ for molded and 3D-printed CT0 samples were the same, but the 3D-printed samples showed a lower value of γFr, which may indicate greater fragility of the 3D-printed gel compared to the molded CT0 gel.

The values of G′ and G″ for the molded CT80 were 6.6 and 5.5 times higher than those for the 3D-printed CT80. Additionally, the molded CT80 had γFr and τFr values 1.4 and 8 times higher than the 3D-printed CT80.

Interestingly, the decrease in the strength of linkage by inclusion of CT into the gel did not appear to be observed if the samples were formed by molding, since parameters G′_LVE_, G*_LVE_, and tan [δ]_AF_ did not differ between the CT0 and CT80-molded gels (Table 5). Furthermore, the addition of CT to the molded samples appeared to increase the number of linkages, but in 3D-printed samples, the number of linkages was lower with the CT80 gel than with the CT0 gel (Table 4). Such parameters as tan [δ]_LVE_ and γL, which are related to the timescale of the junction zone, did not change when adding CT to the ink both when using 3D printing and molding.

## 3. Discussion

Personalized nutrition is one of the most exciting promises of the biotechnological production of plant food raw materials [3,4] and 3D-printing food technology [5,10], since both approaches can be aimed at creating a diet enriched with special health-promoting components for specific population groups. Plant tissue can provide carbohydrates, vitamins, minerals, antioxidants, and other bioactive substances for healthy human nutrition [22]. One innovative approach to address consumer demand is to add various plant-based functional carbohydrates, proteins, lipids, and fibers to 3D-printed food. However, the non-printability of plant tissue significantly limits the development of 3D food inks enriched with plant cell cultures. Therefore, additives must be added to improve the ink’s flow and viscosity in order to create a paste that can be printed. In a previous study, food ink made from 3% k-carrageenan was successfully 3D printed when enriched with the callus tissue at concentrations of 33 g/100 mL, while the ink cured in the printer extruded at higher callus concentrations [12]. Enrichment with plant tissue as much as possible is important to obtain food with beneficial properties. We report here the development of an agar/MP-based ink formulation with a substantially higher callus tissue enrichment (85 vs. 33 g/100 mL) in contrast with the results of our prior findings. We were able to accomplish this while keeping the ink printable.

The study used CT, which was obtained by homogenizing frozen/thawed callus biomass, since non-homogenized callus biomass did not allow 3D printing. Previously, the rheological and mechanical properties of callus tissues had not been determined. Their characterization made it possible to distinguish the rheological parameters that are important for the printability of food raw materials in the process of preparing them for inclusion in ink formulations for 3D printing. It was found that the rheological and mechanical properties of CT were significantly different from those of fresh callus biomass. This was a predicted outcome because the living cells that make up fresh callus determine its structural and physicochemical characteristics. Despite extensive research on callus cultures [23,24,25], the rheological characteristics of callus biomass have not previously been studied. Therefore, it is important to note that fresh callus biomass exhibited strong gel properties. The dynamic moduli G′ and G″ indicate the elastic and viscous properties of the material; thus, values of G′ higher than G″ indicate that the materials demonstrate gel-like behavior [7]. It can be assumed that this is due to turgor pressure in intact cells as well as the gel-forming properties of the intercellular substance. Gel-like properties have been shown previously for Nostoc sphaeroides biomass [26]. Freezing/thawing followed by homogenization weakens the gel structure, but CT also exhibits elastic properties, as could be seen from the values G′ >> G″ and tan [δ] < 1. CT was found to compose a large content of polysaccharides, including pectins. Pectins are known to possess gelling properties in the presence of divalent cations [27]. Therefore, pectins appeared to be partly responsible for the viscoelastic properties of CT, since the presence of calcium and magnesium was found in CT. The presence of the fractions of hardly and easily hydrolysable polysaccharides in lupine callus was previously shown [12], but their composition has not been established. The monosaccharide composition indicated the presence of arabinogalactans and/or galactans, typical of plant callus tissue cultures, and pectic polysaccharides (linear homogalacturonans and/or rhamnogalacturonans).

Understanding food materials’ rheological characteristics is crucial for estimating their printing performance and enhancing their capacity for self-sufficiency when using extrusion-based printing. The agar’s gelling properties played a major role in determining the viscosity of the inks used. Agar is a linear polysaccharide made up of 3,6-anhydro-L-galactose and D-galactose units alternately linked by α-(1→3) and β-(1→4) glycosidic bonds. Galactan chains take a random and stiff coil conformation at high temperature. The coils order to form helices and subsequently aggregate into a three-dimensional network of thick bundles to form a gel upon cooling below the temperature of gelation [13]. It was previously found that the melting and gelling temperatures of agar were ca. 70 and 40 °C, respectively [28]. CT0 ink gel was loaded into the extruder at a temperature of 50 °C, at which the apparent viscosity was about 6 kPa·s. The addition of CT significantly reduced the viscosity of the ink gel (*n* < 1), making it easier for it to flow through the printing nozzle. This effect may be partly due to the presence of free monosaccharides in CT, which have hygroscopicity. In accordance with our results, the study [29] reported that isomaltose reduced the viscosity of agar ink containing Cordyceps powder due to the fact that it has many water-binding sites, carries many water molecules, and acts as a lubricant, thereby reducing the rigidity and tightness of the network structure. The small fractions of lipids in CT may also account for the lubricant effect, as was previously shown for 3D-printed snacks from mushrooms [30]. Rheological properties including yield stress (τ0), flow behavior index (K) and flow characteristic index (*n*) were assumed to correspond to extrudability [31]. It has initially been suggested that the yield stress of a food gel should be between 500 and 1500 Pa to achieve suitable printability [32]. The data obtained did not confirm this assumption, since the τ0 values for CT0, CT80, CT85, and CT75 were significantly lower than the specified range. Data on τ0 value and printability were collected and analyzed by Outrequin et al. [9]. It was found that some gels with lower τ0 values than the proposed range could still be extruded. In particular, this was observed for products using shiitake mushrooms [33] and mashed potatoes [34] by 3D printing with various gum additions, including arabic gum, xanthan gum, and k-carrageenan gum. The mentioned above [33] ink, containing shiitake powder and arabic gum, xanthan gum, or k-carrageenan, was found to be harder to extrude at a value of K higher than 2242 kPa·s. In our investigation, the ink exhibited acceptable extrudability at significantly higher K values. The decrease in the K value as a result of the addition of CT to agar/MP ink is consistent with the data of the study [35], in which replacing a fraction of the biopolymer content with mango puree decreased the K values. Our data confirm the assumption that no universal values have been defined regardless of the type of materials used to yield ink with good printability. It is important to note that printer parameters such as pressure, flow rate, and the nozzle size also affect extrusion.

Furthermore, the storage modulus G′ is thought to be a crucial factor in determining how stable the 3D-printed structure’s shape is [9]. At a fixed nozzle diameter, a 3D structure with a greater storage modulus may tolerate more stress before collapsing than a structure with a lower value. It was previously reported that food gels with G′ and G″ values between 100 and 10,000 Pa can be correctly printed [36]. However, 3D printing using CT as the only ink ingredient has not been successful. Acceptable 3D printability was shown using the ink formulation, which was obtained using MP and a 3% agar solution (CT0). CT0 was characterized by a more elastic behavior than CT, demonstrating G′ and G″ values in the range of 6000–43,000 Pa. MP is widely used in 3D food printing, as it has good extrusion ability and load-bearing capacity without deformation [18,20,21]. At the same time, there are a few studies in which agar is used as an ink component for 3D printing [19]. The high G′ for the CT0 was possibly due to the aggregation of agarose molecules that resulted in the rigid structure. The study of [18] showed that agar increased the thixotropy and yield stress of potato puree, apparently forming a strong gelling network. The authors demonstrated easily hand-able post-printing, a precise, definite dimension of layers, great resistance to compressed deformation, and the high stability of a structured product made from 1% agar plus potato puree. However, poor fluidity, retarded extrusion, and non-continuous flow of this material were also indicated. In the present study, inks containing MP less than 15 or more than 30 g/100 mL did not have 3D printability. The joint use of agar and MP made it possible to reproduce a number of characteristics (hardness, G′, G″, tan [δ], k′, k″, and z) of fresh callus biomass in a CT0 3D-printed gel. However, CT0 ink did not allow reproducing elasticity, γL, τL, τFr, and γFr values. 

The addition of CT significantly reduced the G′ value in our study. This is not consistent with the data of Kim and co-workers [37], who showed that adding vegetable powder to the hydrocolloid solutions increased G′ values. This indicates the peculiarities of the rheological properties of CT in comparison with the properties of vegetable and fruit pastes, which are widely used to create 3D products. It was also proposed that the loss tangent (tan δ) is a useful metric to characterize the shape stability of printed objects as an alternative to using G′ to define stability. Samples with a higher tan δ were shown by Azam and colleagues to be non-self-supporting, which led to poor form stability. According to their findings, the range of acceptable tan δ values was 0.238 to 0.290. The sample still showed proper shape stability below this range; however, some of them were difficult to extrude through the nozzle due to their increased elasticity, which resulted in a rough surface. On the other hand, samples that were outside of this range seemed to have incorrect shape holding for tan δ beyond this range [38]. The range of tan δ should be wider and should be between 0.052 ≤ tan δ ≤ 0.268, as suggested by Gholamipour-Shirazi et al. [39], which is in line with the tan δ values obtained in the present study (0.14–0.19). A comparable range of tan δ values was also discovered in the literature about the 3D printing of food gels that comprised potato starch, skimmed milk, icing sugar, and cream [40] or sodium alginate, soy protein isolate, and gelatin [41].

The relationship between the complex modulus G* and the applied oscillation frequency ω can be described by a power-law equation (G*(ω) = A × ω1/z) [42]. The 3D samples printed with CT0 and CT80 inks had high z-values, which indicates the network extension related to the number of rheological units interacting within the network, and high A values, which indicates the strength of these interactions. The resulting A and z values (15,000–42,000 and 9–21, respectively) show more interaction forces within the network and a board network extension than those, for example, in the structure of chocolate (11–125 and 1–2, respectively) [42].

It should be noted that most studies on the development of food materials for extrusion printing use rheological characteristics to predict the printability of that material. In our approach, rheological analysis revealed the influence of CT on factors that determine the structure of an already 3D-printed gel, such as strength of linkage, number of linkages, and timescale of junction zone.

In addition to products enriched with plant material, specially shaped foods are considered promising for personalized nutrition. Therefore, it is important to evaluate how new printing materials enable precise food deposition and shaping. It was possible to fabricate 3D-printed constructs with good stability using a narrow concentration range of CT (70–85 g/100 mL). The accuracy of printing using CT-containing ink was 85–96% and did not differ significantly from that using CT0 ink. We assessed the accuracy of printing based on the geometric dimensions of the 2D-printed grids, although the error percentage compared to an ideal printing area was not calculated for 1D- and 3D-printed shapes. However, CT-containing inks made it possible to print a cubic structure, which was a 3D version of the 2D lattice, and 3D rounded structures. In previous published papers, solid cylinders have been a frequently employed test shape [36]. In a number of investigations, a 2D lattice [43,44] and a 3D cube with internal cavities [45] were also printed to demonstrate the printability of the ink. Several shapes and structures have been designed and printed to improve the appeal of food, including objects like an anchor, gecko, snowflake, ring, tetrahedron heart shape, bear head, Chinese characters, and many others [46]. The data obtained indicate that CT can be included in the composition of MP and agar-containing inks intended for 3D printing of gels with a shape complexity. 

The mechanical profiles obtained clearly demonstrated the influence of CT on the properties of the 3D-printed gels. Hydrogen bonding is well known to be the primary mechanism involved in agarose gelation [13]. Therefore, the decrease in hardness of CT-containing 3D-printed gel may suggest that CT interferes with the formation of hydrogen bonds between agar molecules. This assumption is consistent with the data showing that the CT80 3D-printed gel demonstrated lower values of elasticity and fracture stress (τFr), which were assumed to relate to the number of linkages in the hydrocolloids [47]. In addition, CT appeared to reduce the strength of the linkage, which can be estimated using values of G’LVE, G*LVE, and tan [δ]AF [47]. However, the type of these linkages was not investigated in the present study. 

The rheological and mechanical properties of molded gels were then investigated to determine the effect of 3D printing procedure on gel formation. The molded samples had relatively lower rheological parameters linked to the type of linkage, the stabilizing ability (strength of linkage), and the extent of network stiffness (number of linkages). This could be due to the fact that the thin jet of ink (3 mm in diameter) extruded from the printer nozzle cooled and solidified faster than the ink material poured into the mold. These data were consistent with textural analysis data. Indeed, during the puncture test, the molded gel broke at a lower probe penetration depth than at the penetration of the 3D-printed sample, indicating that the molded gel was more brittle. In addition, the 3D-printing procedure decreased the timescale of the junction zone, i.e., the time required for the transformation of hydrogel network chains to the thermodynamically ideal state. An unexpected result was a decrease in hardness and Young’s modulus under the influence of the 3D-printing procedure. This result can be explained by the layered structure of the 3D-printed gel. It is likely that the hardness of the entire layered object is reduced due to the mechanical properties at the contact point between two adjacent food layers. Opposite results were obtained in the study [48], which compared the properties of 3D-printed and molded carrots with the addition of guar gum, xanthan gum, and gelatin. The hardness of 3D-printed samples was higher. Interestingly, the effect on the gel properties of the 3D printing procedure with CT-containing ink differed from that using CT-free ink. The CT80 3D-printed gels seemed to have a lower strength of linkage and a smaller number of linkages than the CT80 molded samples, although the mechanism of interaction of CT with other ink components during their gelation requires further study. Generally, a comparison of 3D-printed and molded samples revealed the effect of the 3D printing procedure on the properties of the manufactured food product. These data indicate that the use of 3D printing expands the possibilities of obtaining texture-modified products from the same food paste.

## 4. Conclusions

In this study, food ink made from 3% agar and MP (15–25 g/100 mL) was successfully enriched with lupin CT at concentrations of 75–85 g/100 mL, and the CT-containing food ink was shown to be 3D printable. The use of agar and MP in parent ink formulation CT0 reproduced partly the rheological and mechanical properties of fresh callus biomass. The accuracy of 3D printing using CT-containing ink did not differ significantly from that using CT-free ink. Thus, the present study demonstrates that CT may be added to ink in order to enrich food gel with plant cell material and enable the 3D printing of specially shaped food gels.

## 5. Materials and Methods

### 5.1. Preparation and Characterizing CT

A callus culture of lupine (*Lupinus angustifolius* L.) was cultivated at 26 °C in the dark for 21 days on Murashige–Skoog medium containing vitamins (mg/L: B1—1.0; B2—0.5; B3—2.0; B5—1.0; B6—1.0; B7—1.0; B9—0.5; B12—0.0015) and phytohormones (mg/L: naphthylacetic acid—1.0; kinetin—0.1). A freshly harvested callus was microscopically examined using a Levenhuk Zoom&Joy D870 T optical microscope with a Levenhuk D800 T Camera video eyepiece.

Fresh callus biomass was frozen at −20 °C to obtain frozen/thawed callus biomass. Frozen callus biomass was thawed and passed through a 1 mm metal sieve to remove cell aggregates to obtain homogenized CT.

The thermogravimetric method, the Barnstein’s method, and the Folch method were used to determine the dry matter content, protein content, and free lipids in the CT. 

The gas chromatography-mass spectrometry (GLC-MS) of trimethylsilyl ethers of sugars was used to identify and quantify free monosaccharides in the carbohydrate content of soluble dry components of callus. The GLC-MS of sugars was carried out on a G2589A gas chromatograph (Agilent Tech., Santa Clara, CA, USA) with an HP-5MS capillary column (0.25 mm × 30 m) (Hewlett-Packard, Palo Alto, CA, USA) and a 5973 INERT mass spectrometer (Agilent Tech., Santa Clara, CA, USA). The quantitative determination of sugars was carried out using the internal standard method. To calculate the glucose and fructose content, calibration graphs for D-glucose and D-fructose (0.10–1.00 mg/mL) were used.

To determine the content of polysaccharides, freeze-dried CT (11.37 g) was treated with a boiling mixture of chloroform and methyl alcohol (2:1 by volume) to inactivate enzymes and remove low-molecular-weight compounds. The isolation of water-soluble polysaccharides from defatted material was carried out by sequential exhaustive extraction with distilled water at 68 °C and then with a 0.7% aqueous solution of ammonium oxalate at 68 °C after treating the remainder of the material with a dilute solution of HCl (50 °C, 1 h) at pH 3.8–4.0. The completeness of polysaccharide extraction was controlled by a qualitative reaction according to Smith’s method. The extracts were evaporated and dialyzed, and the polysaccharides were precipitated by adding a 4-fold volume of 96% ethyl alcohol. The precipitate was dissolved in distilled water and freeze dried. As a result, two fractions of the water-soluble polysaccharides LA-1 (373.0 mg) and LA-2 (472.8 mg) were obtained.

### 5.2. Rheological Characterization 

The strain and frequency sweep measurements were performed using a rotational-type rheometer (Anton Paar, Physica MCR 302, Graz, Austria) with a parallel plate geometry (diameter 25 mm; gap 4.0 mm). A controlled shear rate mode was used to evaluate the strain sweep from 0.01 to 100% of the strain amplitudes at a constant frequency and stress of 1 Hz at 20 °C. The following parameters G′, G″, tan δ, G*, γL, τL, τFP, G*FP, τFr, and tan δAF were determined as described earlier [47]. Using the power law equation, the shear strain dependence of G′ and G′ was ascertained.

The mechanical spectra obtained from the frequency sweep experiments were described by G′ and G″ (Pa) values as a function of frequency in the range of 0.3–70.0 Hz at 20 °C and a constant stress of 9.0 Pa [49]. The power law function [50] was expressed as follows:

η = K_c_ × *y^n^*, (1)

where η is the steady viscosity, K_c_ is the consistency constant, *y* is the shear rate, and *n* is the power law index or flow behavior index.

The degrees of frequency dependence for G′ and G″ were determined by the power law parameters described in [51]. The strength of the network (A, Pa·s^1/z^) and the network extension parameter (z) were evaluated according to [52]. Temperature sweeps were carried out from 50 to 5 °C at a rate of 5.0 °C/min [53].

### 5.3. The 3D-Printing Process

The frozen callus biomass was thawed and then run through a metal sieve with a 1 mm mesh size to remove cell aggregates in order to produce CT. Three ink formulations, designated CT80, CT85, and CT75, were prepared based on 80, 85, and 75 g of CT, respectively; the CT0 formulation was prepared without CT (Table 6).

The agar powder (3 g/100 mL) was dispersed in distilled water, which was then agitated and heated to 98.6 °C for 40 min in a Biosan WB-4MS water bath. Then, MP dried powder was added, and the mixture was left for 15 min in a water bath, after which the solution was cooled to 50 °C and mixed with CT. Printing was carried out using a ZMORPH FAB 3D printer (ZMorph S.A., Wrocław, Poland). A 3 mm nozzle was used. Cube-shaped samples with dimensions of 2 × 2 × 2 cm (width, length, and height) were printed individually for rheological and textural studies. Models for printing were created in the Voxelizer for Fab program (version 3.0.0), which is a software bundle for the ZMORPH FAB 3D printer (ZMorph S.A., Wrocław, Poland).

In a separate experiment, ink was poured into cube-shaped silicone molds measuring 2 × 2 × 2 cm (width, length, and height) to prepare molded gels.

### 5.4. Measurement of Mechanical Properties

Samples with a height of 1 cm were used for the puncture test, which was carried out on a TA-XT Plus texture analyzer (Texture Technologies Corp., Stable Micro Systems, Godalming, UK) equipped with a probe diameter of 2 mm. The test was carried out at room temperature. Hardness, which indicates the strength of a gel, was measured as the maximum peak force at any time through the puncture [54]. Young’s modulus indicates the stiffness of a material and is measured as the relationship between stress (force per unit area) and strain (proportional deformation) in a material in the linear elasticity regime of a uniaxial deformation [55]. Young’s modulus was calculated using the following equation: E = (F/A)/(DH/H), where F is the force (N) measured during compression, A is the cross-sectional area of the gel sample, and DH/H is the uniaxial deformation Elasticity, which indicates the capability of gels to recover their original shape after large deformations, was measured as the traveling distance of the probe that penetrated from the start of compression to a break peak. A short distance of penetration indicates a brittle gel, whereas a long distance of penetration indicates a more elastic gel [56].

### 5.5. Scanning Electron Microscopy

The surface morphology was examined using a scanning electron microscope (SEM) (JEOL, JSM6510LV, Peabody, MA, USA) at 10 kV as described earlier [12]. 

### 5.6. Statistical Analysis

The significance of the differences among the means was estimated with one-way ANOVA. Statistical differences with *p*-values lower than 0.05 were considered significant. All calculations were performed using the statistical package Statistica 10.0 (StatSoft, Inc., Tulsa, OK, USA). The data presented were expressed as the means ± SD.

## Figures and Tables

**Table 1:** General characterization of lupin callus biomass.

Callus	Weight (g)	Volume (cm^3^)	Density (g/cm^3^)	MC (%)	pH
Fresh	3.2 ± 0.6	3.6 ± 0.9	0.9 ± 0.1	97.1 ± 1.1	5.98 ± 0.00
Frozen/Thawed	2.0 ± 0.1 *	2.8 ± 0.4 *	0.7 ± 0.1 *	95.2 ± 0.7 *	4.66 ± 0.00 *

The data are presented as the mean ± standard deviation (SD.) *—*p* < 0.05 compared to fresh callus (*n* = 10). MC—moisture content.

**Table 2:** Frequency dependence of viscoelastic parameters for fresh, frozen/thawed callus, and CT: viscosity and frequency (0.3 < ω < 70.0 or at 0.54/1.11/10.50/30.60/50.00 Hz).

Parameters			Fresh	Frozen/Thawed	CT
k′ (Pa·s)			22,464 ± 3032 ^a^	20,734 ± 13,082 ^a^	13,953 ± 1341 ^a^
k″ (Pa·s)			3026 ± 228 ^a^	2881 ± 1729 ^a^	1837 ± 170 ^a^
A			24,273 ± 2760 ^a^	21,058 ± 13,082 ^a^	13,937 ± 1395 ^a^
k″/k′			7.45 ± 0.75 ^a^	7.11 ± 0.51 ^a^	7.59 ± 0.25 ^a^
η*_s_			3727 ± 871 ^a^	3265 ± 1895 ^a^	2187 ± 207 ^a^
*n*’			0.07 ± 0.03 ^a^	0.11 ± 0.02 ^b^	0.11 ± 0.01 ^b^
*n*″			0.06 ± 0.02 ^a^	0.17 ± 0.02 ^b^	0.18 ± 0.01 ^b^
z			21.21 ± 4.95 ^a^	9.31 ± 1.37 ^b^	8.71 ± 0.79 ^b^
Frequency (Hz)	0.54	G′ (kPa)	23,818 ± 3488 ^a^	19,378 ± 13,032 ^a,b^	12,083 ± 2108 ^b^
		G″ (kPa)	7208 ± 2118 ^a^	3847 ± 2534 ^b^	1749 ± 285 ^b^
	1.11	G′ (kPa)	22,872 ± 6283 ^a^	21,329 ± 13,210 ^a^	13,411 ± 2300 ^a^
		G″ (kPa)	3380 ± 620 ^a^	2917 ± 1799 ^a,b^	1753 ± 291 ^b^
	10.50	G′ (kPa)	25,083 ± 2472 ^a^	26,616 ± 16,587 ^a^	16,886 ± 2948 ^a^
		G″ (kPa)	3328 ± 932 ^a^	4075 ± 2550 ^a^	2576 ± 453 ^a^
	30.60	G′ (kPa)	27,458 ± 4922 ^a^	30,178 ± 18,911 ^a^	19,106 ± 3445 ^a^
		G″ (kPa)	3637 ± 1604 ^a^	5216 ± 3474 ^a^	3263 ± 647 ^a^
	50.00	G′ (kPa)	28,846 ± 5543 ^a^	33,179 ± 21,298 ^a^	20,333 ± 3765 ^a^
		G″ (kPa)	3599 ± 872 ^a^	5359 ± 2775 ^a^	3548 ± 794 ^a^

Different letters ^a^ and ^b^ indicate significant (*p* < 0.05) differences between fresh, frozen/thawed callus, and CT (callus tissue). *n* = 6. The frequency dependencies of the elastic (k′ and *n*′), loss (k″ and *n*″), and complex (A and z) moduli; the overall loss tangent (k′/k″); and the slope of complex viscosity (η*_s_).

**Table 3:** Monosaccharide composition * of polysaccharide fractions of lupin callus.

Polysaccharide	UA ^a^	Gal ^a^	Xyl ^a^	Glc ^a^	Rha ^a^	Ara ^a^	OMe ^b^
LA-1	47.3	29.5	2.0	5.0	1.0	13.8	0.7
LA-2	90.9	2.2	0.2	0.6	1.8	4.2	0.8

^a^ and ^b^—data were calculated as mol. % and weight %, respectively. UA—uronic acids, Gal—galactose, Xyl—xylose, Glc—glucose, Rha—rhamnose, Ara—arabinose, OMe—the amount of methyl groups. *—Monosaccharide composition was detected using gas chromatography-mass spectrometry of trimethylsilyl ethers of sugars.

**Table 4:** Mechanical properties of the CT0 and CT80 3D-printed and molded food gels in a puncture test.

Parameters	3D-Printed Samples		Molded Samples	
	CT0	CT80	CT0	CT80
Hardness (N)	0.32 ± 0.03	0.21 ±0.03 ^a^	0.38 ±0.04 ^#^	0.21 ± 0.01 ^b^
Young’s Modulus (kPa)	728 ± 68	511 ± 89 ^a^	1612 ± 124 ^#^	922 ± 57 ^b^
Elasticity (mm)	2.4 ± 0.3	2.8 ±0.9 ^a^	0.85 ± 0.07 ^#^	0.76 ± 0.04 ^b,#^

^a^: differences are significant (*p* < 0.05) between 3D-printed CT80 and 3D-printed CT0; ^b^: differences are significant (*p* < 0.05) between molded CT80 and molded CT0; ^#^: differences are significant (*p* < 0.05) between molded and 3D-printed samples of the same formulation (*n* = 8).

**Table 5:** Dynamic rheological properties of the CT0 and CT80 3D-printed and molded food gels.

Parameters	3D-Printed Sample		Molded Sample	
	CT0	CT80	CT0	CT80
τ0 (Pa)	168 ± 42	30 ± 9 ^a^	400 ± 57 ^#^	331 ± 40 ^#^
K	5116 ± 894	2484 ± 181 ^a^	7351 ± 681 ^#^	7204 ± 315 ^#^
*n*	−0.955	−0.925	−0.913	−0.879
*(A) Strength of linkage*				
G′_LVE_ (Pa)	49,774 ± 3175	6303 ± 605 ^a^	42,518 ± 999	41,630 ± 2703 ^#^
G*_LVE_	50,353 ± 1966	6616 ± 362 ^a^	43,331 ± 926 ^#^	42,441 ± 2118 ^#^
tan [δ]_AF_	0.17 ± 0.01	0.05 ± 0.01 ^a^	0.08 ± 0.04 ^#^	0.05 ± 0.01
*(B) Number of linkages*				
G*max/G*_LVE_	1.06 ± 0.01	1.04 ± 0.02	1.02 ± 0.01 ^#^	1.05 ± 0.01 ^b^
τFr (Pa)	13,241 ± 2329	1621 ± 711 ^a^	7820 ± 1725 ^a,#^	12,969 ± 1510 ^b,#^
*(C) Timescale of junction zone*				
tan [δ]_LVE_	0.14 ± 0.02	0.19 ± 0.06	0.18 ± 0.04	0.15 ± 0.03 ^#^
γL (%)	0.16 ± 0.03	0.24 ± 0.04	0.20 ± 0.05	0.26 ± 0.03
γFr (%)	0.84 ± 0.09	1.34 ± 0.21 ^a^	3.10 ± 0.68 ^#^	1.85 ± 0.34 ^b,#^

^a^: differences are significant (*p* < 0.05) between 3D-printed CT80 and 3D-printed CT0; ^b^: differences are significant (*p* < 0.05) between molded CT80 and molded CT0; ^#^: differences are significant (*p* < 0.05) between molded and 3D-printed samples of the same formulation (*n* = 8).

**Table 6:** Ink formulations based on lupin CT.

Ink Formulation	Agar, g	H_2_O, mL	MP, g	CT, g
CT0	3	100	20	0
CT80			20	80
CT85			15	85
CT75			25	75

## Data Availability

All data and materials are available on request from the corresponding author. The data are not publicly available due to ongoing research using a part of the data.
